# Parental life events cause behavioral difference among offspring: Adult pre-gestational restraint stress reduces anxiety across generations

**DOI:** 10.1038/srep39497

**Published:** 2016-12-21

**Authors:** Nan He, Qiao-Qiao Kong, Jun-Zuo Wang, Shu-Fen Ning, Yi-Long Miao, Hong-Jie Yuan, Shuai Gong, Xiang-Zhong Cui, Chuan-Yong Li, Jing-He Tan

**Affiliations:** 1 College of Animal Science and Veterinary Medicine, Shandong Agricultural University, Tai-an City 271018, P. R. China

## Abstract

While effects of gestational, neonatal or adolescent stress on psychological alterations in progeny have been extensively studied, much less is known regarding the effects of adult pre-gestational life events on offspring behavior. Although full siblings often display behavioral differences, whether the different parental life events prior to different pregnancies contribute to these behavioral differences among siblings is worth studying. In this study, male and female adult mice were restrained for 60 days before mating with unstressed or stressed partners. F1 offspring were examined for anxiety or mated to generate F2. Both F1 females and males from restrained mothers and/or fathers showed significantly reduced anxiety and serum cortisol and increased mRNA levels of glucocorticoid receptor and brain-derived neurotrophic factor compared to control offspring from unstressed parents. Similar behavioral and molecular changes were also observed in F2 females and males. Although restraint of adolescent mice reduced anxiety in F1 of both sexes, social instability of them increased anxiety predominantly in F1 females. Thus, adult pre-gestational restraint reduced offspring’s anxiety across generations; different stressors on parents may cause different phenotypes in offspring; individual behaviors can depend on adult life experiences of parents.

Depression and anxiety disorders are common public health concerns^1^,^2^,^3^. However, the pathophysiological mechanisms that underlie these diseases are poorly understood. Epidemiologic studies indicate that children who had adverse experiences early in life are at increased risk for the development of such disorders in adulthood^4^,^5^. While studies using animal models suggest that gestational^6^,^7^,^8^,^9^,^10^, neonatal^11^,^12^ and adolescent^13^,^14^ stress can cause psychological alterations in the progeny, whether maternal or paternal adult life events prior to pregnancy can evoke behavioral changes in offspring is less well understood^15^. In light of their significant implications for evolutionary biology and disease etiology^16^,^17^, research is needed to document the potential for stress-induced psychological modifications to affect future generations of descendants.

It is not uncommon to notice that siblings born to the same parents differ markedly in certain characteristics. For instance, differences among siblings have been reported in smoking frequency^18^ and self-control capabilities^19^. Mechanisms for inter-sibling differences are largely unknown, although they have traditionally been explained by genetic influences (gene recombination between homologous chromosomes during gametogenesis) and/or by shared and non-shared environmental influences^18^,^19^. Parents experience different cumulative life histories prior to the initiation of each pregnancy. However, the epigenetic influence of parental life events prior to pregnancy on inter-individual difference among descendant offspring has yet to be examined.

Maternal stress hormones such as glucocorticoids that can reach the fetuses through the placenta are one mechanism by which prenatal stress can induce long-lasting alterations upon behavioral and neuron-endocrine systems in the offspring, as has been reported by Barbazanges *et al*.^20^, Weinstock^21^, Maccari *et al*.^22^ and Zagron and Weinstock^23^ for certain context-dependent effects. In fact, the brain is very sensitive to prenatal programming, and an excess of glucocorticoids at critical stages of development can modify neuron-endocrine functions and the morphology of several brain structures^24^. However, humans and animals are challenged with stressors every day, not only for the perinatal period. Stressors that activate the hypothalamic-pituitary-adrenal (HPA) axis were also found to affect oocytes^25^,^26^ and spermatocytes^27^ in adult animals, suggesting that such stressors might cause behavioral alterations in offspring by their direct effects on the developing gametes. Furthermore, epigenetic transgenerational changes in behavior observed in response to gestational stress are difficult to disentangle because both the F1 offspring in utero and their potential F2 germ cells receive exposure.

Although several recent studies suggest that paternal stress before breeding alters offspring HPA axis regulation and behavior in mice^28^ and rats^29^, effects of maternal adult stress prior to pregnancy on offspring behavior have rarely been reported^14^,^30^. Furthermore, while Saavedra-Rodríguez and Feig^13^ reported that chronic social instability during adolescence led to increased anxiety-like behavior of F1 mouse offspring, others observed that paternal stress exposure in newborn mice, throughout puberty or in adulthood resulted in reduced HPA stress axis responsivity^28^ or improved behavioral flexibility^31^ in offspring. Our objectives were (a) to test whether stress of female or male adults prior to mating would cause behavioral alterations in offspring of the subsequent pregnancy and whether such alterations would be trans-generationally transmitted to cause inter-individual differences in the F2 generation; and (b) to determine whether the difference in offspring phenotypes (decreased versus increased anxieties) between the above reported studies was caused by different stress methods or different animal ages when stress was experienced. In the experimental design we adopted, the F0 adult or adolescent mice were exposed to restraint or social instability stress, and the impact of that stress on the germline was examined in F1 and F2 descendants that had not been exposed to the stress.

## Results

### Pre-gestational restraint of adult (F0) mice reduced the anxiety of F1 offspring

**Figure 1:**
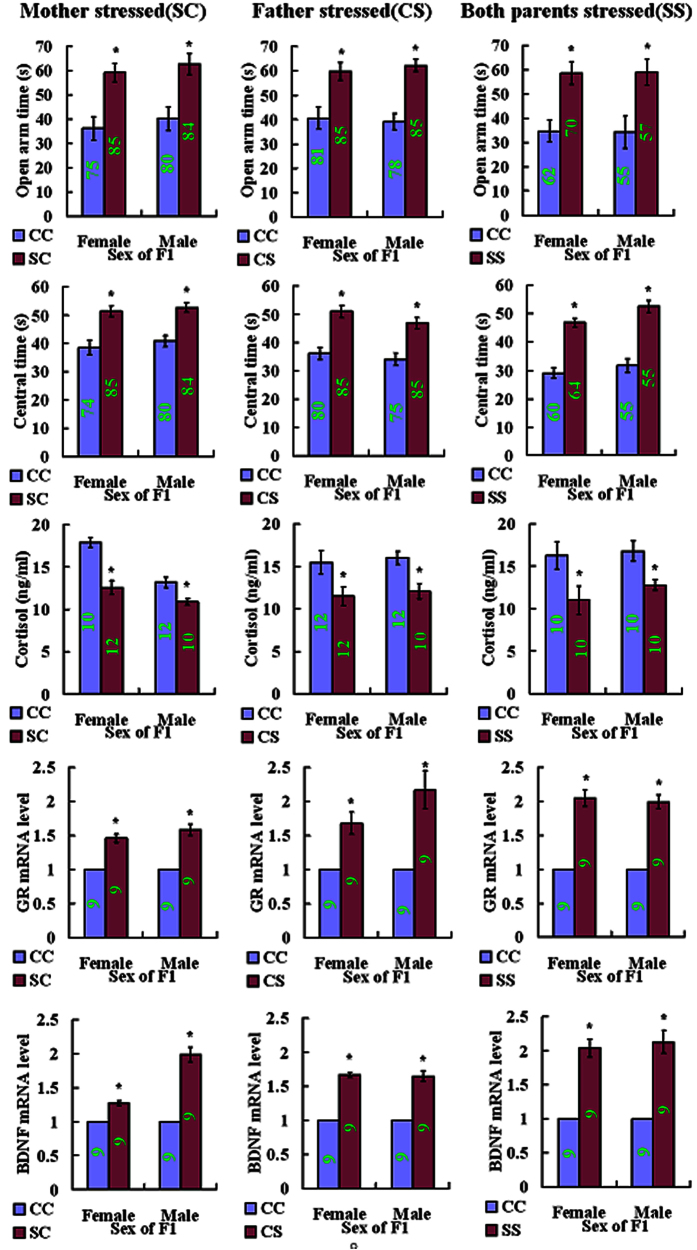
Open arm time of EPM, central area time of OFT, levels of serum cortisol and relative levels of hippocampal GR and BDNF mRNAs in female or male F1 offspring resulting from F0 matings between control father and control mother (CC), stressed mother and control father (SC), control mother and stressed father (CS) or stressed mother and stressed father (SS). For behavior tests, each treatment contained 75–85 F1 offspring from 19–29 litters when either mother or father was stressed, whereas each treatment included 55–70 F1 animals from 14–20 litters when both mother and father were stressed. For serum cortisol assay, each treatment contained 10–12 F1 offspring each from a different litter. For real-time PCR for GR and BDNF mRNAs, each treatment was repeated 3 times with each replicate containing 3 F1 animals each from a different litter. Numbers in each bar indicate the numbers of animals contained in each treatment. *Significant (P < 0.05) difference from CC offspring of the same sex. Whereas data from behavioral tests were analyzed with LMM, data from cortisol assay and real-time PCR were analyzed with Independent-Samples T Test. The P value refers to the fixed effect in the LMM procedure while it refers to the main effect in the Independent-Samples T Test.

Adult male and female mice were restrained for 60 days before being mated with unstressed or stressed partners. On postnatal day 60, the F1 offspring were tested for anxiety-like behavior first by elevated plus-maze (EPM) and then by open field test (OFT) one week later. Offspring from matings between unstressed parents constituted the controls. During EPM, the level of anxiety was quantified by the time spent in the open arm of the maze, and during OFT, stay time in the central area was the measure for predicted anxiety. When either mother (SC) or father (CS) or both parents (SS) were restrained, anxiety of both female and male offspring was reduced significantly, compared to that of control offspring (CC) from unstressed parents (Fig. 1). Thus, both the time spent in the open arm during EPM and the stay time in the central area during OFT were significantly longer in offspring from the stressed parents than in control offspring.

### Pre-gestational restraint of adult F0 mice inhibited the stress-induced increase in glucocorticoid levels while increasing hippocampal mRNA expression of the glucocorticoid receptor (GR) and brain-derived neurotrophic factor (BDNF) of F1 offspring

One week after OFT, one female and one male F1 offspring were randomly selected from one litter and exposed to a 5-min challenge EPM. Immediately following the EPM challenge, the mice were sacrificed to collect blood and hippocampi for assays of serum cortisol and GR and BDNF mRNAs, respectively. Offspring from unstressed parents were also sacrificed to serve as controls. When either mother (SC) or father (CS) was or both parents (SS) were restrained, whereas the levels of serum cortisol in both female and male offspring were significantly lower, the levels of GR and BDNF mRNAs were significantly higher than those in control (CC) offspring produced by unstressed parents (Fig. 1).

### Restraint of F0 mothers for 30 days exerted no significant effect on anxiety and related molecules of F1 offspring

Adult female mice were restrained for 30 days before mating with unstressed male mice (SC). Some unstressed females were mated with unstressed males to serve as controls (CC). Neither behaviors in EPM or OFT nor levels of serum cortisol or hippocampal mRNA expression of GR and BDNF differed significantly between F1 offspring from stressed mothers and control offspring from unstressed parents (Supplementary Fig. S1).

### The F2 offspring derived from matings between F1 mothers and fathers showed significantly reduced anxiety

**Figure 2:**
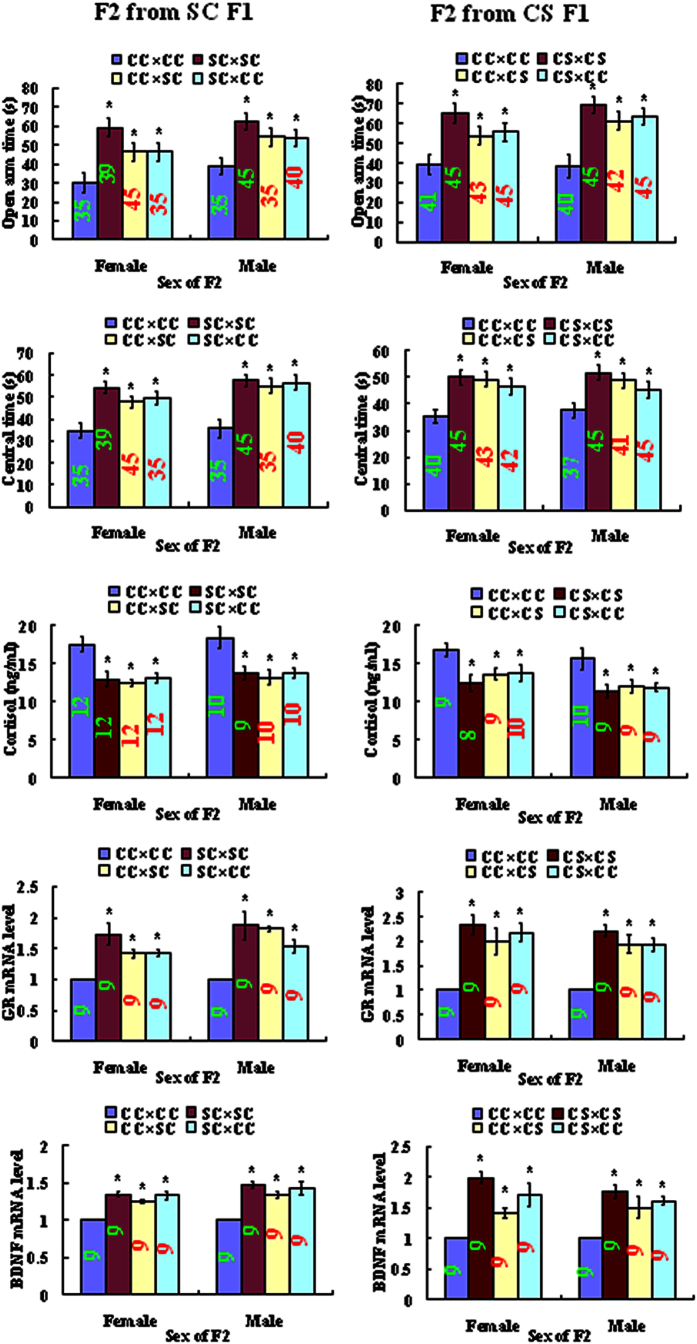
Open arm time of EPM, central area time of OFT, levels of serum cortisol and relative levels of hippocampal GR and BDNF mRNAs in female or male F2 offspring resulting from different combinations between SC or CS F1 mice. For behavioral tests, each treatment contained 35–45 F2 offspring from 10–15 litters. For serum cortisol assay, each treatment contained 9–12 F2 animals each from a different litter. For real-time PCR, each treatment was repeated 3 times with each replicate containing 3 mice each from a different litter. Numbers in each bar indicate the numbers of animals used in each treatment. *Significant (P < 0.05) difference from CC × CC offspring within offspring sex. Whereas data from behavioral tests were analyzed with LMM, data from cortisol assay and real-time PCR were analyzed with ANOVA. The P value refers to the fixed effect in the LMM procedure while it refers to the main effect in ANOVA. The F values and degrees of freedom of ANOVA in female and male offspring in panels for cortisol, GR and BDNF are shown in Supplementary Table S2.

To test whether the reduced anxiety phenotype was retained across generations, F2 offspring were produced from matings among CC, SC or CS F1 animals (Table 1). The resultant F2 offspring were observed for EPM and OFT performance on postnatal day 60. Both F2 females and males from the F1 parental combinations involving either SC or CS F1 mice showed significantly reduced anxiety-like behavior, compared to that observed in control CC × CC mice derived from matings between CC females and CC males. That is, both the open arm time during EPM and the central area time during OFT were significantly longer in the SC × SC, CC × SC, SC × CC, CS × CS, CC × CS or CS × CC F2 mice than those in the CC × CC animals (Fig. 2).

### The F2 offspring showed reduced levels of serum cortisol but increased levels of hippocampal GR and BDNF mRNA expression

One week after OFT, one female and one male F2 offspring were randomly selected from one litter and exposed a 5-min challenge EPM. Immediately following the EPM challenge, the mice were sacrificed to collect blood and hippocampi for assays of serum cortisol and GR and BDNF mRNAs, respectively. Offspring from unstressed parents were also sacrificed to serve as controls. Both F2 females and males from parental combinations involving either SC or CS F1 mice showed significantly reduced cortisol but increased GR and BDNF mRNA levels, compared to those observed in control offspring derived from matings between CC females and males. Thus, whereas the EPM stress-induced cortisol level was lower, levels of GR and BDNF expression were significantly higher in the SC × SC, CC × SC, SC × CC, CS × CS, CC × CS or CS × CC F2 mice than those in the CC × CC animals (Fig. 2).

### Our long-term restraint system stressed animals consistently while having no effects on food and water intake

**Figure 3:**
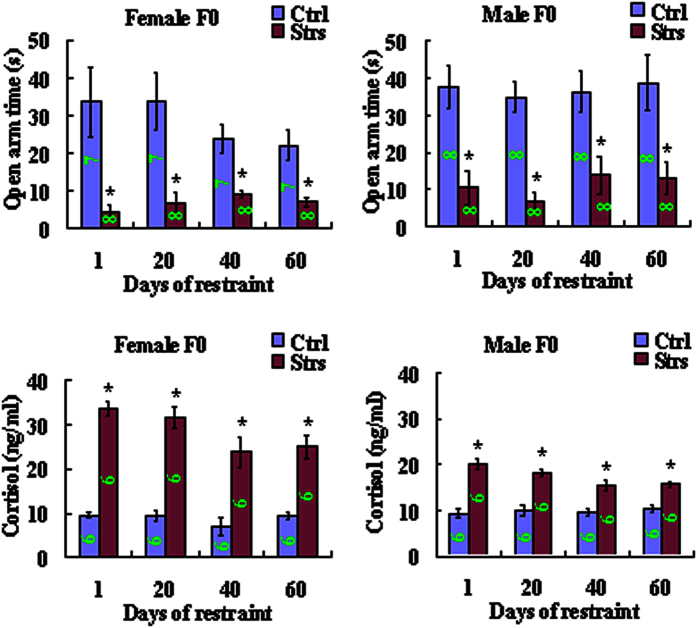
Open arm time and serum cortisol level in female and male control (Ctrl) or female and male stressed (Strs) mice during restraint stress of F0 mice. For behavioral tests, each treatment contained 7–8 mice. For serum cortisol assay, each treatment contained 6 animals each from a different litter. Numbers in each bar indicate the numbers of animals used in each treatment. *Significant (P < 0.05) difference from control mice on the same stress day. Data were analyzed with Independent-Samples T Test, and the P value refers to the main effect.

To test whether our restraint system would affect feeding and whether a food and water deprivation control would be necessary for experiments, food and water intake was compared between restrained F0 and unrestrained control female and male mice on different days of restraint. The results indicate that the average intake of food and water in restrained mice was either not different from, or occasionally higher than, that in unrestrained control mice (Supplementary Table S1). To test the efficacy of our restraint system, serum cortisol concentration and the EPM performance were examined in F0 mice after different times of restraint. At all time points examined, stressed mice showed significantly higher levels of serum cortisol while spending significantly less time in the open arm of EPM than unstressed control mice on the same day of restraint (Fig. 3). Together, the results suggest that (a) our restraint system stressed mice consistently while not affecting feeding; and (b) pre-gestational parental restraint stress reduced anxiety of offspring by activating the parental HPA axis.

### Chronic restraint and social instability during adolescence cause different effects on the anxiety-like behavior and the anxiety-related molecules in F1 offspring

**Figure 4:**
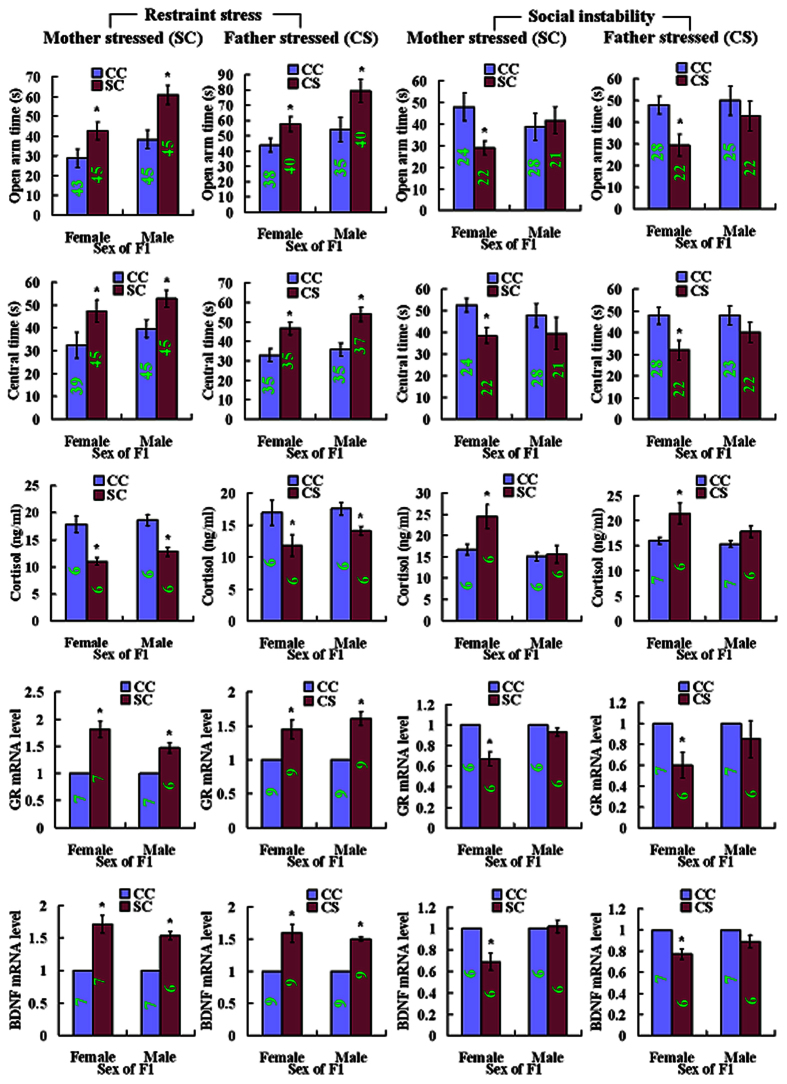
Open arm time of EPM, central area time of OFT, levels of serum cortisol and relative levels of hippocampal GR and BDNF mRNAs in F1 females or males resulting from adolescent F0 matings between control father and control mother (CC), restraint- or social instability-stressed mother and control father (SC), or control mother and stressed father (CS). For behavior tests following restraint stress, each treatment contained 35–45 F1 offspring from 12–16 litters. For behavior tests following social instability stress, each treatment contained 21–28 F1 offspring from 7–9 litters. For serum cortisol assay, each treatment contained 6–7 F1 offspring each from a different litter. For real-time PCR, each treatment was repeated 3 times with each replicate containing 2–3 F1 animals each from a different litter. Numbers in each bar indicate the numbers of animals used in each treatment. *Significant (P < 0.05) difference from CC offspring of the same sex. Whereas data from behavioral tests were analyzed with LMM, data from cortisol assay and real-time PCR were analyzed with Independent-Samples T Test. The P value refers to the fixed effect in the LMM procedure while it refers to the main effect in the Independent-Samples T Test.

It was reported that chronic social instability during adolescence led to increased anxiety-like behavior of F1 mouse offspring^13^. This was different from the present results that chronic restraint during adulthood led to reduced anxiety-like behavior of F1 mouse offspring. To determine whether the difference was caused by different ages when stress was experienced or by different stress methods, we compared the effects of chronic restraint and social instability during adolescence of mice on anxiety behavior and related molecules in their F1 offspring. The results showed that although restraint of adolescent mothers or fathers reduced anxiety in both F1 males and females with increased time in open arm of EPM and central area of OFT, increased levels of hippocampal GR and BDNF mRNAs and decreased levels of serum cortisol (Fig. 4), the social instability stress increased offspring’s anxiety predominantly in F1 females with decreased time in open arm of EPM and central area of OFT, decreased levels of hippocampal GR and BDNF mRNAs and increased levels of serum cortisol compared to F1 from control unstressed parents.

## Discussion

The present results indicate that maternal or paternal adult restraint prior to mating reduced anxiety of resultant offspring. Thus, the female and male F1 offspring from mothers and/or fathers who had been restrained for 60 days showed significantly reduced levels of anxiety-like behavior and of serum cortisol but increased levels of hippocampal GR and BDNF mRNA expression, compared to offspring from unstressed parents. It is known that the hippocampus is the principal target site in the brain for adrenocortical steroids, as it has the highest concentration of GR^32^. There is considerable evidence that the hippocampus modulates most aspects of HPA activity, including basal and peak secretion as well as the onset and termination of responses to stress^33^. Depletion of GR without inducing cell loss in the hippocampus results in corticosterone hypersecretion^34^. Furthermore, whereas mouse strains that under-express GR have a disinhibited HPA system and demonstrate increased helplessness after stress exposure, mouse strains over-expressing GR exhibit reduced helplessness after stress exposure and enhanced HPA feedback regulation^35^,^36^.

BDNF is widely expressed in the adult mammalian brain, the highest levels being found in the hippocampus^37^. In a clinical study, Karege *et al*.^38^ found that serum BDNF levels were significantly lower in patients with major depression than in controls and that severity of depression mainly accounted for a negative correlation between BDNF levels and depression scores. Mice with a variant BDNF (Met/Met) gene showed defective BDNF secretion and when placed in stressful settings, exhibited increased anxiety-related behaviors that were not normalized by an antidepressant^39^. Furthermore, hippocampal BDNF levels have been shown to be reduced in GR(+/−) mice that showed significantly reduced GR expression^35^,^36^. Taken together, the present results suggest that adult pre-gestational restraint reduced offspring stress response by increasing offspring expression of GR and BDNF in the hippocampus, which led to decreased secretion of glucocorticoids.

Studies have shown that mouse and rat offspring that were exposed to gestational prenatal stress show increased fear and anxiety^9^,^40^,^41^, increased and prolonged stress-induced corticosterone secretion^42^,^43^,^44^,^45^,^46^, and decreased numbers of hippocampal corticosteroid receptors^46^,^47^. Furthermore, investigations of Barbazanges *et al*.^20^ and Kapoor *et al*.^48^ demonstrate that impairment induced by gestational prenatal stress on activity of the HPA axis of offspring depends on high levels of maternal corticosterone secretion during restraint stress. Thus, blocking stress-induced corticosterone secretion by adrenalectomy with corticosterone-substitutive treatment suppresses the prolonged stress-induced corticosterone response and the reduced type I hippocampal corticosteroid receptors observed in prenatally stressed adults. The administration of corticosterone to these mothers reinstates the effects induced by the prenatal stress. Furthermore, immobilization of rat dams during pregnancy altered hippocampal synaptic plasticity in young offspring by preventing the proteolytic conversion of pro-BDNF to mature BDNF^49^.

The chronic restraint system adopted in our experiment appeared to stress animals consistently across time: at all time points examined during the 60-day stress period, stressed mice showed significantly higher levels of serum cortisol while spending significantly less time in the open arm of the EPM than did unstressed controls. However, although restraint of mothers for 60 days reduced offspring anxiety significantly, restraint of mothers for 30 days exerted no significant effect. The results suggest that pre-gestational restraint reduced the anxiety of offspring through activating the parental HPA axis, and that an accumulation of HPA products was needed to evoke reduced anxiety in offspring. Although it seems that both pre-gestational and gestational stresses alter offspring behavior by activating the parental HPA axis, the physiological mechanisms two stressors employ may differ. For example, whereas the HPA products from gestational stress can pass through the placenta and act directly on the brain of the offspring^50^, those from pre-gestational stress must influence the offspring indirectly by way of the gametes. In fact, recent studies in mice have shown that paternal stress exposure throughout puberty or in adulthood reduced offspring’s HPA stress axis responsivity via altering sperm microRNA content^28^,^51^.

Whereas offspring from rats stressed during gestation show increased levels of anxiety and depression-like behavior during adulthood^52^, the offspring from females stressed prior to initiation of pregnancy had lower levels of anxiety than controls (the current results). In the gestational stress model, stress of from 7 to 10 days duration was sufficient to induce behavioral changes in the offspring of rats^20^,^49^ or mice^9^. In the pre-gestational stress model used in our study, however, offspring showed significant behavioral changes only after their parents had been stressed for 60 days. Furthermore, whereas marked gender effects on behavior were reported in offspring from gestationally stressed rats^47^,^53^,^54^, no gender differences were observed in F1 offspring from the pre-gestationally stressed adult mice in the present study and in studies reported by Rodgers *et al*.^28^,^51^.

The present results demonstrate that restraint of sires altered offspring behavior in the same way and to the same extent as restraint of dams did. This further confirms that pre-gestational stress might have used mechanisms different from those used by gestational and neonatal stress to affect offspring behavior, because whereas gestational and neonatal stress acted on diploid genome, pre-gestational stress acted on haploid genome of either male or female gametes. It has been reported that pre-reproductive stress on adolescent female rats alters corticotrophin releasing factor type 1 expression in ova^14^, and that robust changes in sperm microRNA content were observed after stress exposure of male mice^28^,^55^. In addition, the results that restraint of sires can reduce offspring anxiety have removed the maternal effects as a source of variation for the pre-gestational stress-induced modifications of offspring behavior. In studies dealing with the gestational and neonatal stress, modified maternal adaptations to pregnancy and altered maternal behavior have often been implicated as the potential mechanisms by which transgenerational programming of the stress response occurs^56^.

A recent study reported that chronic social instability during adolescence and early adulthood of mice led to increased anxiety and social deficits that were transmitted predominantly to female offspring across generations^13^. To determine whether the difference in offspring phenotypes (decreased versus increased anxieties) between the present study and those reported by Saavedra-Rodríguez and Feig^13^ was caused by different stress methods (restraint versus social instability) or different ages when stress was experienced (adult versus adolescence), we compared the effects of chronic restraint and social instability during adolescence of mice on levels of anxiety and related molecules in their F1 offspring. The results show that although social instability increased anxiety predominantly in the F1 female offspring as Saavedra-Rodríguez and Feig^13^ reported, restraint of adolescent mice reduced the anxiety in their offspring of both sexes. Chronic variable stress of male mice throughout puberty or in adulthood reduced HPA stress axis responsivity in offspring of both sexes^28^. Repeated stress of male rats on a elevated platform prior to mating led to a reduction in stress reactivity in male offspring^29^. Chronic unpredictable stress of female rats before pregnancy was associated with high risk of depression in male progeny^30^. Furthermore, restraint of mice during pregnancy led to increased anxiety in offspring^9^. It is thus proposed that different stressors that parents experienced prior to pregnancy evoke different psychological modifications in offspring and that the same stressor may cause different offspring phenotypes when applied to mothers prior to or during pregnancy. It has been reported that the same early life stress can have different consequences on stress reactivity and behavior later in life. For example, maternal separation during postnatal life in mice or rats can increase stress reactivity in some models^57^, but can have opposite effects in other models^58^. In addition, whether animal strain differences between studies could contribute to some differences in results is very worth researching.

In this study, when F1 mice from restrained F0 mice were mated with control mice or with each other, both female and male F2 offspring showed significantly reduced levels of anxiety and serum cortisol and increased levels of GR and BDNF mRNA expression compared to control offspring produced by control mothers and fathers. The results suggest that the reduction in stress response induced by pre-gestational stress of the adult parents was transmitted across generations. Although prenatal stress and postnatal handling of rats have been associated with increased and reduced anxiety, respectively, in the F1 generation^40^, reports on the transgenerational effects of prenatal stress or postnatal handling on HPA function in offspring past the F1 generation are very limited^13^,^56^.

In summary, present results show that adult maternal or paternal chronic restraint stress prior to pregnancy reduces offspring’s anxiety and that modifications were trans-generationally inheritable. Although restraint of adolescent mice reduced anxiety in F1 of both sexes, social instability of adolescent mice increased anxiety predominantly in female F1. The current results have undoubtedly added to the growing list of examples of transgenerational transmission of the effects of stress. The phenomenon that adult maternal or paternal restraint stress prior to pregnancy reduces offspring’s anxiety is of phylogenetic importance and may have an adaptive basis, possibly matching the phenotype of the offspring to conditions it likely would experience after birth. Of great interest is the bold idea that some psychological disorders might be prevented by subjecting parents to chronic mild stressors, leading to alleviated stress responses in the offspring. Furthermore, such studies are important models for research on how an environmental factor can reprogram the germline and promote a transgenerational phenotype, which has significant implications for evolutionary biology and disease etiology. However, although research on the effects of gestational prenatal stress on offspring is revealing some of the underlying mechanisms, those for the effects of pre-gestational parental stress on offspring remain largely unknown, due to the marked differences between the two stressors as discussed above. Studies are under way in this laboratory to explore the mechanisms by which chronic restraint and social instability of adolescent mice produced different phenotypes in F1 and F2 offspring.

## Methods

The experimental procedures were approved by the Animal Care and Use Committee of the Shandong Agricultural University P. R. China (Permit number: SDAUA-2001-0510). The methods were carried out in accordance with the approved guidelines. Unless otherwise specified, all chemicals and reagents were purchased from Sigma Chemical Co. (St. Louis, MO, USA).

### Mice and stress treatment

Mice of the Kunming breed were kept in a room with a constant temperature (22–25 °C) and 14 h/10 h light-dark cycles, the dark beginning at 8:00 pm. Adult and adolescent mice were 8 weeks and 27 days after birth, respectively, at the initiation of the experiment, at which time age- and sex-matched mouse pairs were randomly assigned to the stress treatment or the non-stressed control.

For the restraint treatments, an individual mouse was put in a micro-cage constructed by the authors, which was placed in an ordinary home cage. The micro-cage offered the same photoperiod and controlled temperature as in the home cage. While in the micro-cage, mice could move back and forth to some extent, but they could not turn around. Food and water were provided during the restraint sessions, conducted for 8 h per day (from 8:00 am to 16:00 pm) for 60 days. Control mice remained in their home cages during the time treated mice were stressed. At the end of the 60-day restraint, female mice were housed in groups of 4, whereas male mice were housed singly in the home cage.

The procedures adopted for the social instability stress were those reported previously^59^. Briefly, the cage mates in each cage were changed twice per week for 8 weeks so that four mice from different cages were put together in a new, clean cage. The rotation schedule was randomized to minimize the likelihood of a repeated encounter of the same mice throughout the experiment. Four control mice were housed in each cage always with the same cage mates. At the end of the 8-week treatment, mice were housed in groups of 4 with cage mates from the last change.

### Breeding

For evaluation of the transgenerational effects of stress, breeding pairs were formed 5 days after the end of the chronic restraint or social instability treatment. To produce F1 generation, stressed females and males were mated with other stressed or control mice. In total, 4 types of breeding pairs were formed as followed: (1) composed of both control mice (CC), (2) composed of stressed females and control males (SC), (3) composed of stressed males and control females (CS), and (4) composed of both stressed females and males (SS). To produce F2 generation, F1 females and males from either stressed mother (SC) or stressed father (CS) were mated with other F1 from either stressed parents or control mice. In total, 7 types of breeding pairs were formed as followed: (1) composed of CC females and CC males, (2) composed of SC females and SC males, (3) composed of CC females and SC males, (4) composed of SC females and CC males, (5) composed of CS females and CS males, (6) composed of CC females and CS males, and (7) composed of CS females and CC males.

The offspring of all groups were weaned on postnatal day 21 and left undisturbed until testing at 2 months of age. Only litters of 8–10 pups with approximately half females and half males were kept for the study. Four to five siblings of the same sex were housed in the same cage until testing.

### Elevated plus-maze (EPM) test

The EPM device consisted of two open arms (30 × 6 cm), alternating at right angles with two closed arms (30 × 6 × 15 cm). The central platform delimited by the four arms was 36 cm^2^. The whole maze was elevated 50 cm above the floor. Each animal’s behavior on the maze was recorded via a video camera mounted on the ceiling above the center of the maze. The camera was connected to an Any-maze video tracking motion analysis system (Stoelting, Wood Dale, IL, USA) running on a personal computer. The test room was maintained at 22–25 °C with 50–55 dB white noise continuously provided. The EPM tests were always performed between 9:00 and 11:00 am except for the test that was designed to challenge the animals before blood collection for hormone assays (the challenge EPM), which took place between 2:00 and 4:00 pm. Before the start of the test, naïve mice were individually placed in a rectangular plastic arena for 30 min in order to habituate them to the test environment. To start the test, a mouse was placed on the central platform, facing an open arm, and was allowed to explore the maze for 5 min. Following a four-paw criterion, numbers of entries and time spent in each arm over the total exploration in both open and closed arms were calculated using the Any-maze software. The device was cleaned with 10% ethanol after each trial to effectively remove the scent of the previously tested animal.

### The open field test (OFT)

The open field test was conducted on a black square floor of 50 cm × 50 cm and white walls 45 cm tall. The floor was divided into 25 10 cm × 10 cm squares, of which those 16 squares touching a wall were designated as peripheral area with the 9 remaining squares designated the central area. Each animal’s behavior in the open field was recorded by video camera and analyzed as described above. The test room was maintained at 22–25 °C, and white noise of 50–55 dB was continuously maintained. The test was conducted from 8:00 to 11:00 am. Before the start of the test, mice were individually placed in the test room for 30 min in order to habituate them to the test environment. To start the test, a mouse was placed in the central area and was allowed to explore the open field for 5 min. Then the numbers of central entries, time spent in central area and distance traveled in the open field were calculated using the Any-maze software. The device was cleaned with 10% ethanol after each trial to remove the scent of the previously tested animal.

### Hormone assays

Blood collection for hormone assay was always conducted between 2:00 and 4:00 pm to avoid the effect of the circadian rhythm. Blood collection from restrained F0 mice was completed immediately after their release from restraint and that from the F1 and F2 mice was conducted after an EPM challenge test. Mice were sacrificed by decollation, and trunk blood (about 1 ml) was collected into ice-cooled centrifugal tubes. After collection, the blood samples were centrifuged (1700 × g, 10 min) at 4 °C to separate serum. The serum collected was stored at −80 °C until hormone assay. Serum cortisol levels were measured by radioimmunoassay at the Central Hospital of Tai-An City using commercial kits from Wei-Fang (3 V) Bioengineering Co. Ltd., Wei-Fang City, P. R. China. The minimum level of detection was 1.0 ng/ml, and the intra- and inter-assay CVs were 5.3% and 6.7%, respectively.

### Quantitative real-time PCR

Hippocampi were recovered at the same time when blood collection was conducted as described above. Hippocampi homogenization was performed using Trizol reagent (1 ml for 50–100 mg hippocampi tissue). Isolated RNAs were re-suspended in diethyl pyrocarbonate-treated MilliQ water (DEPC-dH_2_O) and digested with RNase-free DNase I (Takara Biotechniques, Dalian, China). The purified RNA was dissolved in DEPC-dH_2_O and spectroscopically quantified at 260 nm. Purity and integrity of the RNA was assessed by determination of the A_260_/A_280_ ratio (1.8–2.0) and electrophoresis in 1% agarose.

Reverse transcription was performed in a total volume of 20 μl using Superscript III^TM^ Reverse Transcriptase (Invitrogen Australia Pty. Ltd). Briefly, 2 μl of each RNA sample were mixed in a 0.2 ml reaction tube with 4 μl of dNTP, 1.5 μl Oligo dT_18_ (Takara) and 6 μl of DEPC-dH_2_O, and the mixture was incubated in a PCR instrument at 65 °C for 5 min. As soon as the incubation ended, the reaction tube was cooled on ice for 2 min and then centrifuged (200 × g for 10 sec at 4 °C) for a few seconds. Then, 4 μl of 5× RT buffer, 0.5 μl RNase inhibitor and 0.5 μl Superscript III Reverse Transcriptase were added to the reaction tube. The mixture was then incubated at 50 °C for 1 h, followed by incubation at 70 °C for 15 min before storing at −20 °C until use.

Gene-specific primers for real-time RT-PCR are as follows. For GR, forward 5′-AGT CAA GGT TTC TGC GT -3′, reverse 5′-CCA TCA CTT TTG TTT CG -3′; for BDNF, forward 5′-GCC TCC TCT ACT CTT TCTG-3′, reverse 5′-GGA TTA CAC TTG GTC TCGT-3′; and for Gapdh, forward 5′-AAA CCR GCC AAG TAT GAT GA-3′, reverse 5′-GTG GTC CAG GGT TTC TTA CT-3′. Quantification of mRNA was conducted using the Mx3005 P real-time PCR instrument (Stratagene, Valencia, CA). Amplification reactions were performed in a 10-μl reaction volume containing 1 μl of cDNA, 5 μl of 2× SYBR Green Master Mix (Stratagene), 0.15 μl of ROX (reference dye), 3.05 μl of RNase-free water, and 0.4 μl each of forward and reverse gene-specific primers (10 μM). Cycle amplification conditions comprised an initial denaturation step at 95 °C for 10 min followed by 40 cycles at 95 °C for 5 sec and at 60 °C for 20 sec. Immediately after amplification, PCR products were analyzed by sequencing, dissociation curve analysis and gel electrophoresis to determine specificity of the reaction. Gene expression was normalized to the gapdh internal control. All values were then expressed relative to calibrator samples using the 2^−(ΔΔCT)^ method.

### Measurements of food and water intake

To measure food and water intake, the restrained and control mice were individually in cages with the floor covered by a pressboard. Food (including that crushed on the floor) and water were weighed both before and after treatments. The food and water intake was calculated by subtracting the post-stress weight from the pre-stress weight.

### Data analysis

The behavioral data of the F1 and F2 offspring were analyzed by the Linear Mixed Models (LMM) procedure, and the software used was the Statistics Package for Social Science (SPSS 20.0; SPSS Inc., Chicago, IL, USA). Our data in F1 and F2 offspring are longitudinal (parents to offspring) data within which litter effect is nested. For longitudinal data, traditional analysis method that usually overlooks the litter effect would increase the probability of false positive errors; taking the average of data in each litter could lose a huge amount of sample information, causing false negative errors. The LMM procedure analyzes both the fixed (main) effects (parental stress effects) and the random effects (litter effects). Random effects are incorporated to accommodate among-subject variation^60^. In this study, behavioral data of the F1 and F2 offspring were analyzed using LMM with parental treatment as a fixed factor and litter as a random factor. The covariance structure used was the UN (unstructured) structure. The molecular data and data from F0 animals were arc sine transformed and analyzed either with ANOVA when 3 or more sets of data were involved, or with Independent-Samples T Test when only two sets of data were involved; a Duncan multiple comparison test was used to locate differences. The software used was SPSS 11.5. Data were expressed as mean ± S.E.M. and P < 0.05 was considered significant. The P value refers to the fixed effect in the LMM procedure while it refers to the main (treatment) effect in ANOVA and Independent-Samples T Test.

## Additional Information

**How to cite this article**: He, N. *et al*. Parental life events cause behavioral difference among offspring: Adult pre-gestational restraint stress reduces anxiety across generations. *Sci. Rep.* **6**, 39497; doi: 10.1038/srep39497 (2016).

**Publisher's note:** Springer Nature remains neutral with regard to jurisdictional claims in published maps and institutional affiliations.

## Supplementary Material

Supplementary Information

## Figures and Tables

**Table 1:** The F1 mating types used to produce F2 offspring.

F0 treatment	F1 offspring	F1 combinations	F2 offspring
Females stressed	SC	CC♀ × CC♂	CC × CC
		SC♀ × SC♂	SC × SC
		CC♀ × SC♂	CC × SC
		SC♀ × CC♂	SC × CC
Males stressed	CS	CC♀ × CC♂	CC × CC
		CS♀ × CS♂	CS × CS
		CC♀ ×CS♂	CC × CS
		CS♀ × CC♂	CS × CC

Notes: The table shows the F1 mating types and the resultant F2 offspring. When F0 females were stressed (SC), CC × CC F2 were produced from F1 CC females × CC males, SC × SC F2 were produced from F1 SC females × SC males, CC × SC F2 were produced from F1 CC females × SC males, and SC × CC F2 were produced from F1 SC females × CC males. When F0 males were stressed (CS), CS × CS F2 were produced from F1 CS females × CS males, CC × CS F2 were produced from F1 CC females × CS males, CS × CC F2 were produced from F1 CS females × CC males.
