# Supplementary material for: Parental life events cause behavioral difference among offspring: Adult pre-gestational restraint stress reduces anxiety across generations
Source: Sci Rep. 2016 Dec 21;6:39497. doi: 10.1038/srep39497 (PMC5175168; doi:10.1038/srep39497)
Supplement: Supplementary Information [file srep39497-s1.pdf]

Supplementary information

Title: Parental life events cause behavioral difference among offspring: Adult pre-gestational restraint stress reduces anxiety across generations

Nan He[1,2], Qiao-Qiao Kong[1,2], Jun-Zuo Wang[1,2], Shu-Fen Ning[1,2], Yi-Long Miao[1,2], Hong-Jie Yuan[1], Shuai Gong[1], Xiang-Zhong Cui[1], Chuan-Yong Li[1], and Jing-He Tan[1,3]

1. College of Animal Science and Veterinary Medicine, Shandong Agricultural University, Tai-an City 271018, P. R. China

2. These authors contributed equally to the work.

3. Corresponding author: Jing-He Tan, College of Animal Science and Veterinary Medicine, Shandong Agricultural University, Tai-an City 271018, Shandong Province, P R China; Phone: 0538-8249616; FAX: 0538-8241419; Email: tanjh@sdau.edu.cn

Supplementary figure and table legends

Supplementary Fig. S1. Open arm time of EPM, central area time of OFT, concentrations of serum cortisol and relative levels of hippocampal GR and BDNF mRNAs in female or male F1 offspring resulting from F0 matings between control mother and control father (CC) or between mother stressed for 30 days and control father (SC). For behavior tests, each treatment contained 40-55 F1 offspring from 10-14 litters. For serum cortisol assay, each treatment contained 6 F1 animals each from a different litter. For real-time PCR for GR and BDNF mRNAs, each treatment was repeated 3 times with each replicate containing 4 F1 animals each from a different litter. Numbers in each bar indicate the numbers of animals used in each treatment. # Insignificant ($P>0.05$) difference from CC offspring of the same sex. Whereas data from behavioral tests were analyzed with LMM, data from cortisol assay and real-time PCR were analyzed with Independent-Samples T Test. The P value refers to the fixed effect in the LMM procedure while it refers to the main effect in the Independent-Samples T Test.

Supplementary Table S1. Food and water intake in female (fCtrl) and male (mCtrl) control or female (fStrs) and male (mStrs) stressed animals during restraint stress of F0 mice. Each treatment included 7-8 F0 animals. *Significant ($P<0.05$) difference from control animals within the same column. Data were analyzed with Independent-Samples T Test, and the P value refers to the main effect.

Supplementary Table S2. The F values and degrees of freedom of ANOVA in female and male offspring in panels for cortisol, GR and BDNF of Fig. 2.

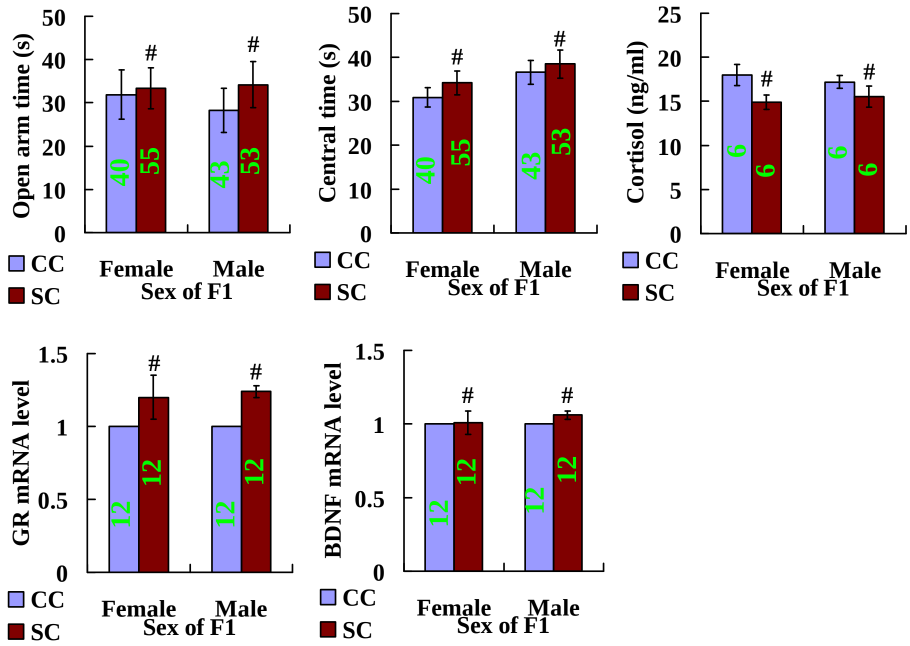

Supplementary Fig. S1. Open arm time of EPM, central area time of OFT, concentrations of serum cortisol and relative levels of hippocampal GR and BDNF mRNAs in female or male F1 offspring resulting from F0 matings between control mother and control father (CC) or between mother stressed for 30 days and control father (SC). For behavior tests, each treatment contained 40-55 F1 offspring from 10-14 litters. For serum cortisol assay, each treatment contained 6 F1 animals each from a different litter. For real-time PCR for GR and BDNF mRNAs, each treatment was repeated 3 times with each replicate containing 4 F1 animals each from a different litter. Numbers in each bar indicate the numbers of animals used in each treatment. # Insignificant ($P>0.05$) difference from CC offspring of the same sex. Whereas data from behavioral tests were analyzed with LMM, data from cortisol assay and real-time PCR were analyzed with Independent-Samples T Test. The P value refers to the fixed effect in the LMM procedure while it refers to the main effect in the Independent-Samples T Test.

Supplementary Table S1. Food and water intake in female (fCtrl) and male (mCtrl) control or female (fStrs) and male (mStrs) stressed animals during restraint stress of F0 mice.

Treatment	Food intake (g)						
fCtrl	0.65±0.03	0.75±0.09	0.76±0.29	0.89±0.37	0.36±0.05	0.31±0.02	0.32±0.01
fStrs	0.64±0.13	0.98±0.06	1.12±0.10	1.10±0.02	0.86±0.10*	0.70±0.03*	0.72±0.05*
mCtrl	1.01±0.14	0.97±0.05	0.92±0.03	1.16±0.02	0.95±0.15	0.85±0.02	1.01±0.11
mStrs	0.94±0.05	1.02±0.67	1.08±0.05	1.14±0.15	1.06±0.14	1.07±0.08	0.90±0.15
Treatment	Water intake (g)						
fCtrl	0.58±0.12	0.80±0.16	0.63±0.16	0.78±0.14	0.71±0.15	0.64±0.08	0.52±0.05
fStrs	0.63±0.12	0.69±0.13	0.71±0.28	0.54±0.16	0.67±0.06	0.55±0.08	0.73±0.26
mCtrl	1.01±0.12	0.90±0.09	1.09±0.12	1.10±0.09	1.07±0.09	0.78±0.12	1.02±0.13
mStrs	1.06±0.11	0.87±0.06	1.10±0.07	1.26±0.03	1.06±0.12	0.79±0.02	0.87±0.08
Restraint days	1-3	9-11	19-21	29-31	39-41	49-51	58-60

Each treatment included 7-8 F0 animals. *Significant ($P<0.05$) difference from control animals within the same column. Data were analyzed with Independent-Samples T Test, and the P value refers to the main effect.

Supplementary Table S2. The F values and degrees of freedom of ANOVA in female and male offspring in panels for cortisol, GR and BDNF of Fig. 2.

	F2 from SC F1		F2 from CS F1	
Sex of F2	Female	Male	Female	Male
Cortisol	F(3,44)=8.18	F(3,35)=7.16	F(3,32)=4.08	F(3,33)=3.68
GR mRNA	F(3,32)=7.32	F(3,32)=13.36	F(3,32)=14.43	F(3,32)=20.75
BDNF mRNA	F(3,32)=16.65	F(3,32)=10.76	F(3,32)=7.84	F(3,32)=12.70

The two numbers in each bracket indicate df1 (degree of freedom between groups) and df2 (degree of freedom within groups), respectively.
